# Glutaminase 1 regulates the release of extracellular vesicles during neuroinflammation through key metabolic intermediate alpha-ketoglutarate

**DOI:** 10.1186/s12974-018-1120-x

**Published:** 2018-03-14

**Authors:** Beiqing Wu, Jianhui Liu, Runze Zhao, Yuju Li, Justin Peer, Alexander L. Braun, Lixia Zhao, Yi Wang, Zenghan Tong, Yunlong Huang, Jialin C. Zheng

**Affiliations:** 1 Center for Translational Neurodegeneration and Regenerative Therapy, Shanghai Tenth People’s Hospital affiliated to Tongji University School of Medicine, Shanghai, China; 2 Department of Pharmacology and Experimental Neuroscience, University of Nebraska Medical Center, Omaha, NE 68198 USA; 3 Department of Pathology and Microbiology, University of Nebraska Medical Center, Omaha, NE 68198-5930 USA

**Keywords:** Glutamine metabolism, α-Ketoglutarate, Extracellular vesicles, HIV-1, Inflammation

## Abstract

**Background:**

Extracellular vesicles (EVs) are important in the intercellular communication of the central nervous system, and their release is increased during neuroinflammation. Our previous data demonstrated an increased release of EVs during HIV-1 infection and immune activation in glial cells. However, the molecular mechanism by which infection and inflammation increase EV release remains unknown. In the current study, we investigated the role of glutaminase 1 (GLS1)-mediated glutaminolysis and the production of a key metabolic intermediate α-ketoglutarate on EV release.

**Methods:**

Human monocyte-derived macrophage primary cultures and a BV2 microglia cell line were used to represent the innate immune cells in the CNS. Transmission electron microscopy, nanoparticle tracking analysis, and Western blots were used to determine the EV regulation. GLS1 overexpression was performed using an adenovirus vector in vitro and transgenic mouse models in vivo. Data were evaluated statistically by ANOVA, followed by the Bonferroni post-test for paired observations.

**Results:**

Our data revealed an increased release of EVs in GLS1-overexpressing HeLa cells. In HIV-1-infected macrophages and immune-activated microglia BV2 cells, treatment with bis-2-(5-phenylacetamido-1,2,4-thiadiazol-2-yl)ethyl sulfide (BPTES) or CB839, two specific GLS inhibitors, significantly decreased EV release, suggesting a critical role of GLS1 in EV release. Furthermore, addition of α-ketoglutarate or ceramide rescued EV release during BPTES treatment, implicating α-ketoglutarate and ceramide as critical downstream effectors for GLS inhibitors. These findings were further corroborated with the investigation of brain tissues in GLS1-transgenic mice. The EV levels were significantly higher in GLS1 transgenic mice than those in control mice, suggesting that GLS1 increases EV release in vivo.

**Conclusions:**

These findings suggest that GLS1-mediated glutaminolysis and its downstream production of α-ketoglutarate are essential in regulating EV release during HIV-1 infection and immune activation. These new mechanistic regulations may help understand how glutamine metabolism shapes EV biogenesis and release during neuroinflammation.

**Electronic supplementary material:**

The online version of this article (10.1186/s12974-018-1120-x) contains supplementary material, which is available to authorized users.

## Background

Extracellular vesicles (EVs) are secretory vesicles budded from the plasma membrane of a variety of cells. EVs range from 50 nm to 1 mm and differ in their origins, either from direct fusion with plasma membrane or intracellular multivesicular bodies. EVs have been detected at an elevated level in the cerebral spinal fluid in patients with mild to severe Alzheimer’s disease, Parkinson’s disease, prion disease, and amyotrophic lateral sclerosis [1, 2]. Protein markers of EVs are present in neuritic plaques in AD brains. Furthermore, increasing EV release likely contributes to the toxicity of amyloid beta and tau phosphorylation [3]. Therefore, EVs play a potential role in the pathogenesis of AD. Mechanisms regulating EV release remain poorly understood. Recent studies indicate that the formation and secretion of EVs are largely dependent on the proper function of ceramide, which is a type of sphingolipids catalyzed by neutral sphingomyelinase (nSMase) from sphingomyelin. More specifically, EV release can be blocked by the inhibition of the nSMase pathway [4, 5]. GW4869, a nSMase inhibitor, significantly reduces the release of EVs and corresponding neurotoxicity in the HIV-infected, as well as immune-activated macrophages and microglia, and in AD models in vitro and in vivo [3, 6, 7].

Glutamine is the most abundant amino acid in the plasma, and the metabolism of glutamine involves hydrolysis to glutamate by mitochondrial enzyme glutaminase 1 (GLS1). Subsequently, glutamate can be excreted or can be further metabolized to α-ketoglutarate. Our previous studies uncovered a pathogenic role of glutaminase (GLS) in neuroinflammation and neuronal injury. There are two major types of GLS identified in mammals, the “kidney-type” glutaminase (GLS1) and “liver-type” glutaminase. GLS1 is the predominant glutamine-utilizing enzyme in the central nervous system (CNS), where “liver-type” glutaminase is expressed at a lower level [8]. GLS1 is known to be associated with cancer cell research and CNS diseases [9–11]. Due to tissue-specific alternative splicing from the same gene, two isoforms of GLS1 were identified in the human brain, kidney-type glutaminase (KGA) and glutaminase C (GAC). Both KGA and GAC appear to catalyze glutamine deamination with comparable enzyme efficacies and kinetics [12, 13]. GAC is upregulated in HIV-associated dementia brain samples and is also released in the conditioned medium from HIV-1-infected macrophages. Furthermore, mitochondrial stress during HIV infection leads to membrane destabilization and release of GLS from the mitochondrial matrix to the cytosol through the permeability transition pore [14, 15].

GLS1 has been identified as an important metabolic factor controlling EV release from astrocytes during neuroinflammation [16]. However, the mechanism by which GLS1 regulates EV biogenesis and release remains unknown. In this study, we determine the mechanism of EV biogenesis and release through GLS1-mediated glutamine metabolism in human macrophages. We further identify two key downstream metabolites-α-ketoglutarate and ceramide-as critical factors regulating EV release during HIV-1 infection and immune activation. These studies may help understand how glutamine metabolism regulates EV release in the context of infection and inflammation.

## Results

### GLS1 overexpression increases EV release in vitro

To further study the functional impacts of GLS1 on EV release, we constructed adenovirus vectors that overexpressed KGA or GAC to mimic the upregulation of these isoforms during HIV-1 infection. The extracellular levels of glutamate increased significantly in both KGA- and GAC-overexpressing HeLa cells at the multiplicities of infection (MOI) of 200 compared with those in uninfected or vector-treated cells (Additional file 1: Figure S1A). An MOI of 200 was then used for the following GLS1-overexpression experiments. The overexpression of KGA and GAC was confirmed by Western blot (Additional file 1: Figure S1B). GLS1 enzyme activity assay confirmed the increase in KGA and GAC activity in protein lysates from KGA- and GAC-overexpressing HeLa cells, suggesting that the overexpressed KGA and GAC were functional (Additional file 1: Figure S1C). Consistent with GLS1 enzyme activity, the levels of intracellular glutamate (Additional file 1: Figure S1D) and extracellular glutamate (Additional file 1: Figure S1E) were increased after KGA and GAC overexpression compared with those in the GFP and control groups.

**Fig. 1:**
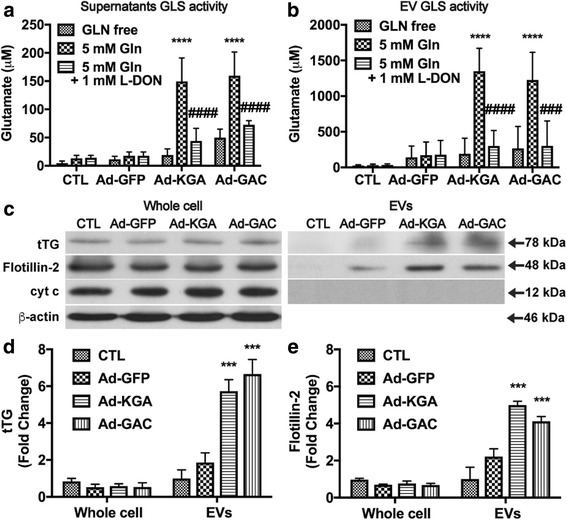
KGA and GAC overexpression increase EV release in vitro. **a** Cell-free supernatants from control (CTL), Ad-GFP-, Ad-KGA-, and Ad-GAC-infected HeLa cells were incubated with or without 5 mM glutamine (Gln) and 1 mM L-DON ex vivo for 2 days. The glutamate levels were determined by RP-HPLC. **b** EVs were isolated from cell-free supernatants from four different groups of HeLa cells and incubated with or without 5 mM glutamine (Gln) and 1 mM L-DON ex vivo for 2 days. Glutamate production from EVs was determined by RP-HPLC. **c** Protein lysates were prepared from the whole cell lysates and EV pellets. The levels of EVs markers, tTG and flotillin-2, were analyzed by Western blot. EV protein loading was normalized with protein concentrations in whole cell lysates. Mitochondrial marker, cytochrome c, was used as a control to exclude contamination of EV pellets. **d**, **e** Densitometric quantifications of the protein levels in EVs were presented as fold change relative to that in control EV lysate. The protein levels in the whole cell lysates were presented as fold change relative to β-actin in the whole cells. Western blot results shown are representative of three independent experiments. CTL, control. Quantification results were normalized to the control group; ANOVA and post-test were performed on the remaining groups. *** and **** denote *p* < 0.001 and 0.0001, respectively, compared with the Ad-GFP group. ^###^ and ^####^ denote *p* < 0.001 and 0.0001, respectively, compared with the 5 mM Gln group, *n* = 3 per group

After confirming KGA and GAC overexpression, we investigated GLS1 activities in the extracellular fluid. We found that cell-free supernatants showed increased glutamate production after the addition of glutamine, indicating that GLS1 overexpression leads to elevated levels of GLS1 activities in the extracellular fluid. L-DON, a GLS1 inhibitor, blocked the excess generation of glutamate in the supernatants, confirming that the observed GLS1 activity in the extracellular supernatants was from the protein GLS1 (Fig. 1a). To confirm that the observed GLS1 activity in the cell-free supernatants was from EVs, we isolated EVs from cell-free supernatants and tested the EVs in GLS1 activity assay. EVs that were isolated from KGA- or GAC-overexpressing HeLa cells generated significantly higher levels of GLS1 when incubated with glutamine (Fig. 1b). When L-DON was added to the enzyme reactions, the generation of glutamate was blocked, suggesting that KGA and GAC overexpression induced EV release that contains high levels of GLS1 activities (Fig. 1b). To determine the EV regulation after KGA and GAC overexpression, proteins from EVs were subjected to Western blot for EV markers, including tTG and flotillin-2. The levels of tTG and flotillin-2 did not change in the whole cell lysates but specifically increased in EVs collected from KGA- and GAC-overexpressed HeLa cells (Fig. 1c–f). The mitochondrial protein cytochrome c was absent in the EV preparation, suggesting that the preparation was not contaminated with subcellular organelles such as mitochondria. Together, these data suggest that elevation of GLS1 is sufficient to induce the extracellular release of EVs.

### EV release in HIV-1-infected macrophages is dependent on glutamine metabolism

**Fig. 2:**
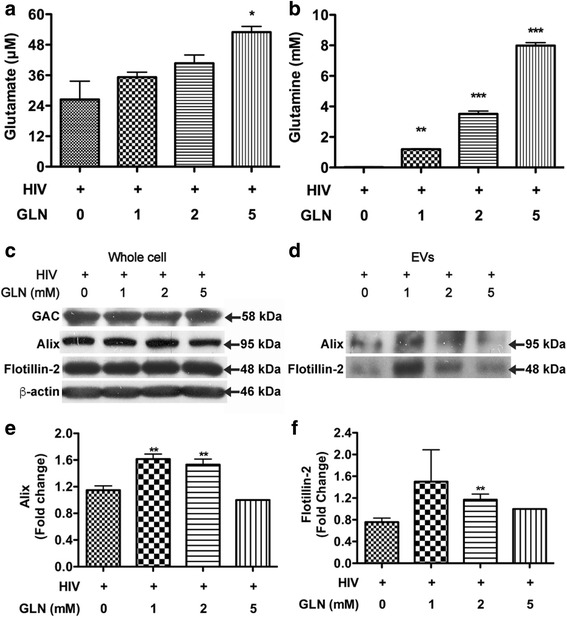
EV release in HIV-1-infected macrophages is dependent on glutamine metabolism. MDM was infected by HIV-1 virus for 6 days, and the medium was changed into serum-free glutamine-free DMEM. Additional glutamine was added to the medium at the concentrations of 1, 2, and 5 mM. **a**, **b** Supernatants were collected and centrifuged at 1500 rpm for 5 min to remove cells. Samples were prepared for RP-HPLC, and the levels of glutamate and glutamine were determined. **c**, **d** Protein lysates were prepared from the whole cell lysates (**c**) and EVs pellets (**d**). The levels of EVs markers Alix and flotillin-2 in EVs, as well as the levels of β-actin in the whole cells, were determined by Western blot. EV protein loading was normalized with protein concentrations in the whole cell lysates. **e**, **f** Densitometric quantifications of the protein levels of EV markers were presented as fold changes relative to that in mock-infected control EV lysates. Western blot results shown are representative of the three independent experiments. Quantification results were normalized to the glutamine 5 mM group; ANOVA and post-test were performed on the remaining groups. *, **, and *** denote *p* < 0.05, 0.01, and 0.001, respectively, compared with that of the control microglia cells, *n* = 3 per group.

Our previous work demonstrated the role of GLS1 in the regulation of EV release [17]. However, the mechanism of how GLS1 regulates the release remains unclear. To determine whether glutamine metabolism is essential for EV release, we added different concentrations of glutamine to HIV-1-infected macrophages for 1 day and then isolated EVs from the cell-free supernatants. The EVs were first subjected to negative staining TEM for characterization. EVs manifested typical morphologies in TEM and had sizes ranged from 40 to 300 nm (Additional file 1: Figure S2A–D). Quantification of EVs showed a significant increase of EVs at a concentration of 1 mM and higher concentrations of glutamine did not appear to further increase EV release (Additional file 1: Figure S2E). Next, the addition of glutamine was confirmed with measurements of glutamate and glutamine in cell-free supernatants by reversed-phase high-performance liquid chromatography (RP-HPLC, Fig. 2a, b). To determine the EV regulation after glutamine treatment, we collected protein lysates from both the whole cells and EVs. The expression levels of GAC under different concentrations of glutamine did not change compared to those in control group (Fig. 2c). EV markers Alix and flotillin-2 did not change in whole cell lysates but specifically increased in glutamine treatment groups at 1 and 2 mM compared to controls (Fig. 2c–f). Glutamine treatment at 5 mM did not induce significantly higher levels of EV release compared with the control. Together, these results suggest that EV release in HIV-1-infected macrophages is dependent on glutamine metabolism.

### EV release in immune-activated microglia is dependent on glutamine metabolism

**Fig. 3:**
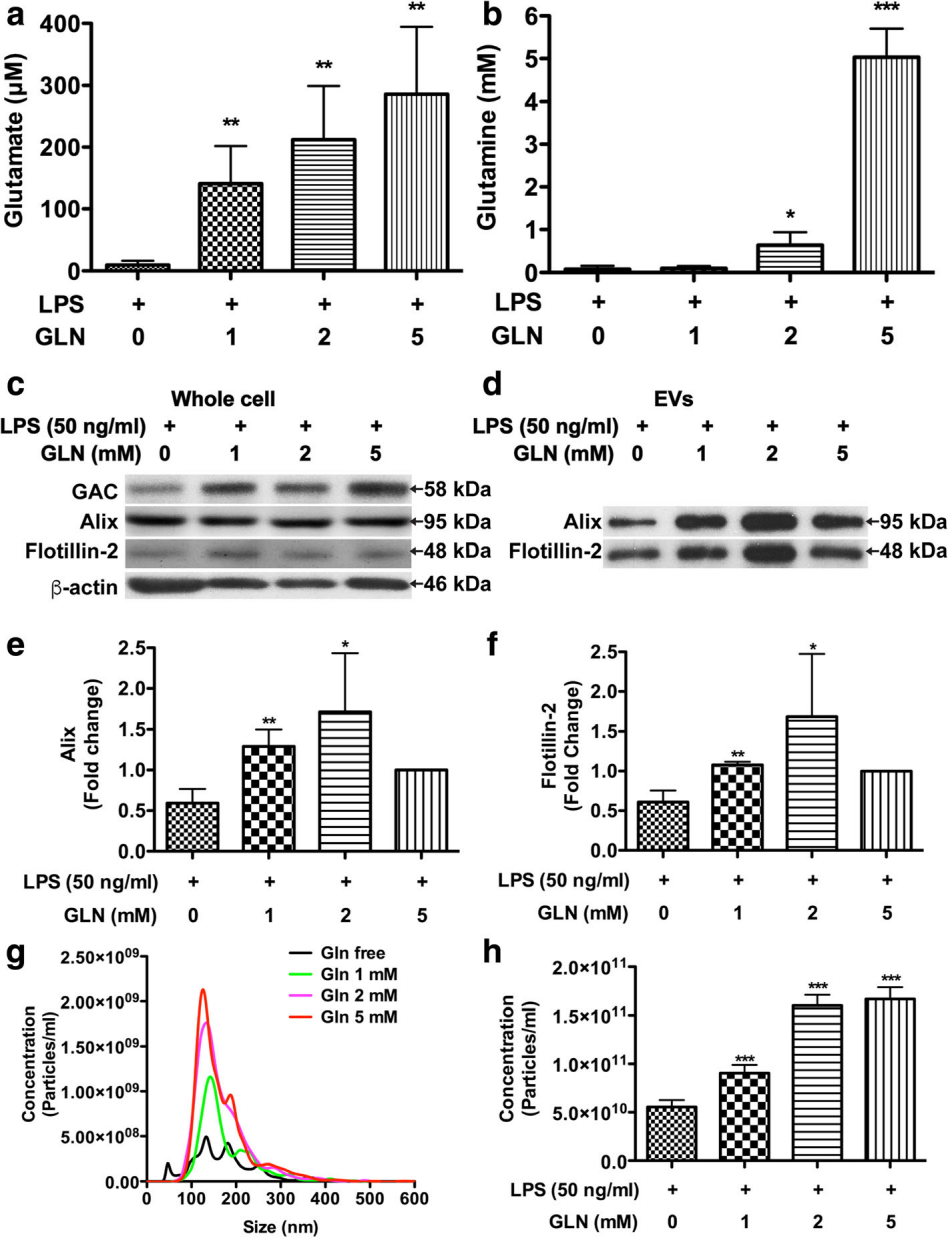
EV release in immune-activated microglia is dependent on glutamine metabolism. When BV2 medium was changed into serum-free glutamine-free DMEM, additional glutamine was added to the medium at concentrations of 1, 2, and 5 mM 6 h prior to LPS treatment. **a**, **b** Supernatants were collected and centrifuged at 1500 rpm for 5 min to remove cells. Samples were prepared for RP-HPLC, and the levels of glutamate and glutamine were determined. **c**, **d** Protein lysates were prepared from the whole cell lysates and EVs pellets. The levels of EVs markers Alix and flotillin-2 in EVs, as well as the levels of GAC and β-actin in whole cells, were determined by Western blot. EVs protein loading was normalized with protein concentrations in the whole cell lysates. **e**, **f** Densitometric quantifications of the protein levels in EV markers were presented as fold changes relative to that in mock-infected control EV lysates. Western blot results shown are representative of the three independent experiments. Quantification results were normalized to the glutamine 5 mM group; ANOVA and post-test were performed on the remaining groups. *, **, ***, and **** denote *p* < 0.05, 0.01, 0.001, and 0.0001, respectively, compared with that of control Gln-free-treated microglia cells, *n* = 3 per group (**a**–**f**). **g** EVs were isolated from culture supernatants and visualized through NanoSight. **h** Quantifications of NanoSight NTA of vesicle concentration for samples from LPS-treated BV2 cell with different glutamine concentrations. ANOVA and Bonferroni post-test; *, **, ***, and **** denote *p* < 0.05, 0.01, 0.001, and 0.0001, respectively, compared with that of control Gln-free-treated microglia cells, *n* = 5 per group

To investigate whether EV release is dependent on glutamine in microglia, a BV2 microglial cell line was used. Different concentrations of glutamine were added to the cultures 6 h prior to lipopolysaccharide (LPS) treatment. After overnight treatment, the levels of extracellular glutamate were increased with the addition of glutamine in a dose-dependent manner (Fig. 3a, b). To determine the EV regulation, protein lysates from whole cells and EVs were subjected to Western blots. In the whole cells, GAC showed a trend of increase with the addition of glutamine concentrations, whereas EV markers remained unchanged (Fig. 3c). In EV lysates, EV markers Alix and flotillin-2 were both significantly increased with the addition of glutamine (Fig. 3d–f). Consistent with Western blot analysis, nanoparticle tracking analysis (NTA) showed that addition of glutamine significantly increased EV release in a dose-dependent manner (Fig. 3g, h). Altogether, these results demonstrated that EV release in immune-activated microglia is dependent on glutamine.

### Inhibition of GLS1 activity reduces EV release in HIV-1-infected macrophages and immune-activated microglia

**Fig. 4:**
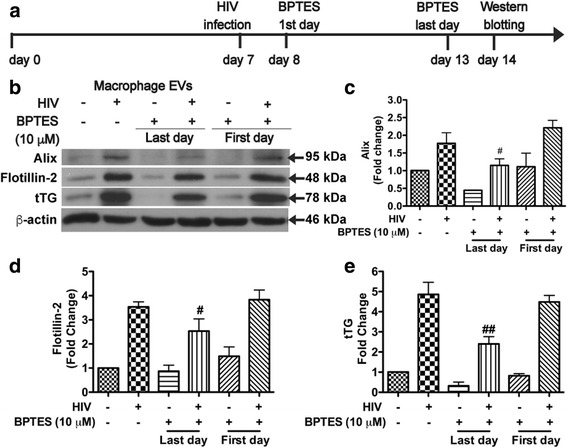
Inhibition of glutaminase reduces EV release in HIV-1-infected macrophages. **a** Timeline of treatments of macrophages prior to collection. **b** EVs were isolated from mock-infected or HIV-1-infected macrophages treated with 10 μM BPTES at the day after infection or 1 day prior to EV isolation. EV protein loading was normalized with protein concentrations in the whole cell lysates. EV markers, Alix, flotillin-2, and tTG in EVs, as well as the levels of β-actin in whole cells, were determined by Western blot. **c**–**e** Densitometric quantifications of the protein levels of Alix, flotillin-2, and tTG in EV markers were presented as fold change relative to that in mock-infected control EV lysates. Western blot results shown are representative of the three independent experiments. Quantification results were normalized to the control uninfected macrophage group; ANOVA and post-test were performed on the remaining groups. * and ** denote *p* < 0.05 and *p* < 0.01, respectively, compared with the HIV-1-infected macrophages, *n* = 3 per group

To further investigate whether GLS1-mediated glutamine metabolism is crucial for EV release in HIV-1-infected macrophages and immune-activated microglia, we used BPTES, a potent GLS1 inhibitor [18]. Human macrophages were infected with HIV-1 virus with 10 μM BPTES prior to sample collections (Fig. 4a). When BPTES was added 1 day prior to EV isolation, EV lysates showed significantly decreased levels of EV markers Alix, Flotillin-2, and tTG compared with those from HIV-1-infected macrophages (Fig. 4b–e). In contrast, when BPTES was added immediately after the HIV-1 infection, the EV regulation was not affected, indicating that BPTES affects the EV release within a limited time frame.

**Fig. 5:**
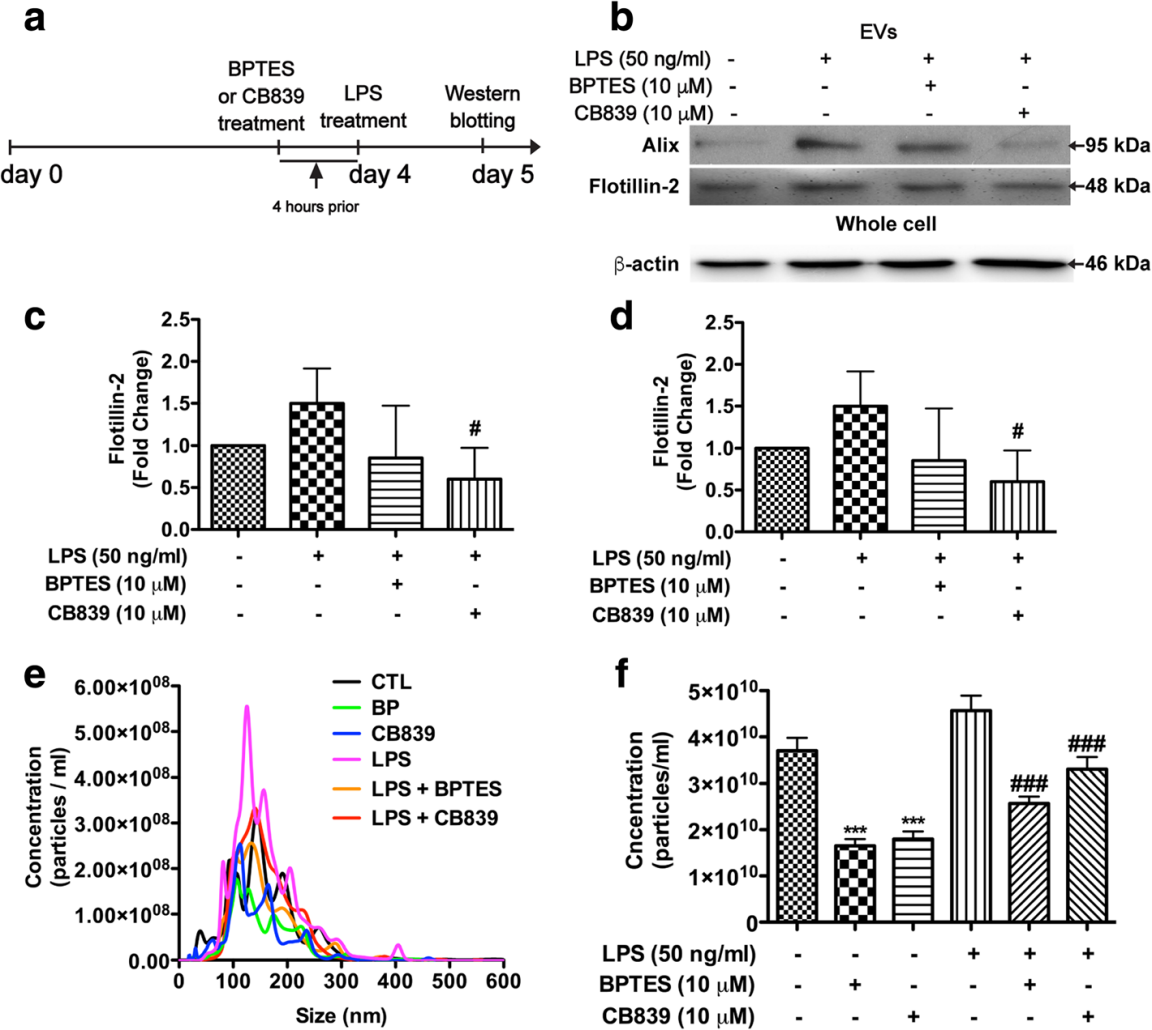
Inhibition of glutaminase reduces EV release in immune-activated microglia. **a** BV2 cells were treated with 10 μM BPTES or 10 μM CB839 4 h prior to LPS treatment overnight. BV2 cell regular medium was changed to serum-free DMEM medium when GLS1 inhibitors were added. **b** Protein lysates were prepared from EV pellets in control, LPS-treated, and LPS-treated with GLS1 inhibitor BV2 cells. The levels of EVs markers Alix and flotillin-2 in EVs, as well as the levels of β-actin in whole cells, were determined by Western blot. **c**, **d** Densitometric quantification of the protein level of GAC in the whole cell lysates was presented as fold change relative to densitometric quantifications of the protein levels of in EV markers were presented as fold change relative to that in control EV lysates. Quantification results were normalized to the unactivated control BV2 group; ANOVA and post-test were performed on the remaining groups. ^#^ denotes *p* < 0.05 compared with that of LPS-activated microglia group, *n* = 3 per group. **e** EVs were isolated from culture supernatants and visualized through NanoSight. **f** Quantifications of the NanoSight NTA of vesicle concentration for samples from control and LPS-treated BV2 cells with or without BPTES and CB839. ANOVA and Bonferroni post-test; *** denotes *p* < 0.0001 compared with that of the control BV2 cells. ^###^ denotes *p* < 0.0001 compared with that of the LPS-treated BV2 cells, *n* = 5 per group

To investigate GLS1-mediated glutamine metabolism in immune-activated microglia, we used BPTES and CB839, both of which are potent GLS1 inhibitors [19]. BPTES or CB839 was added to BV2 cells prior to LPS treatment, and EV regulation was determined through Western blot and NTA (Fig. 5a). LPS, BPTES, and CB839 treatment did not significantly change BV2 cell viability (Additional file 1: Figure S3). In contrast, both levels of Alix and flotillin-2 were significantly increased after LPS stimulation in the EVs isolated from BV2 cells, suggesting that LPS increases EV release in BV2 cells (Fig. 5b–d). Pretreatment with BPTES and CB839 reduced the levels of Alix and flotillin-2 in LPS-activated BV2 cells (Fig. 5b–d). Consistent with Western blot data, NTA revealed that treatment with BPTES and CB839 significantly reduced the EV particles in both the LPS-activated and unactivated control BV2 cells. Collectively, these results indicated that the release of EVs in HIV-1-infected macrophages and LPS-treated microglia is dependent upon GLS1-mediated glutamine metabolism.

### EV release in immune-activated microglia is dependent on the production of α-ketoglutarate

**Fig. 6:**
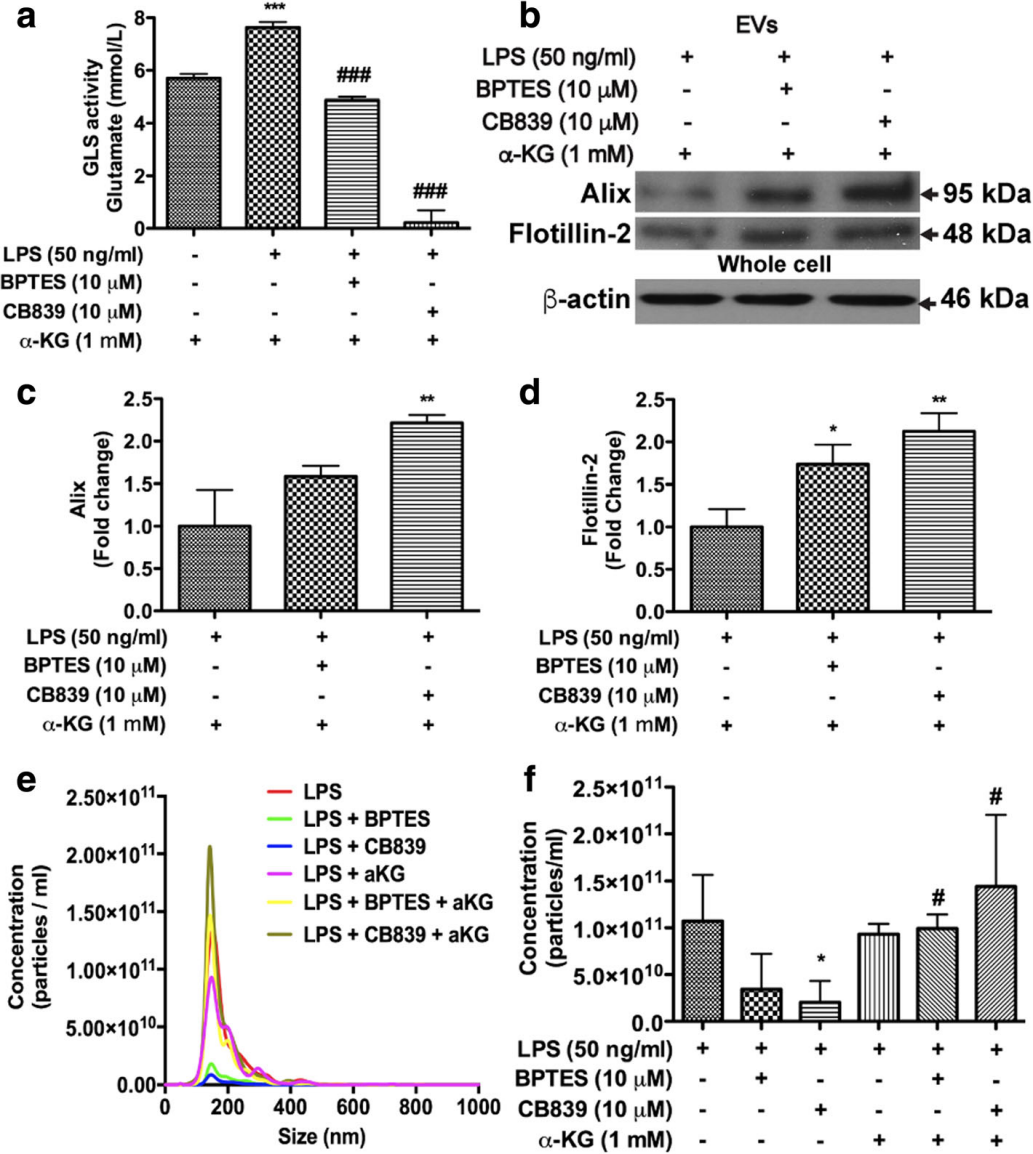
EV release in immune-activated microglia is associated with the level of α-ketoglutarate. BV2 cells were treated with 10 μM BPTES or 10 μM CB839 4 h prior to LPS treatment overnight. BV2 cell regular medium was changed to serum-free DMEM medium when GLS1 inhibitors were added. Two hours prior to LPS treatment, 1 mM of α-KG was added to BV2 cells. **a** Protein lysates were collected from LPS-treated BV2 cells with or without GLS1 inhibitors or α-KG. GLS1 activities were determined by the enzyme activity assay. **b** Protein lysates were prepared from EV pellets. The levels of EVs markers Alix and flotillin-2 in EVs, as well as the levels of β-actin in whole cells, were determined by Western blot. EV protein loading was normalized with protein concentrations in the whole cell lysates. **c**, **d** Densitometric quantifications of the protein levels of in EV markers were presented as fold change relative to that in mock-infected control EV lysates. ANOVA and Bonferroni post-test; * and ** denote *p* < 0.05 and 0.01, respectively, compared with that of the control microglia cells, *n* = 3 per group. **e** After 1 mM of α-KG treatment, NTA was conducted with 100× dilution of collected EVs with filtered PBS. **f** Quantifications of the NanoSight NTA of vesicle concentration for samples from LPS-treated BV2 cell with GLS1 inhibitors and α-KG. ANOVA and Bonferroni post-test; * denotes *p* < 0.05 compared with that of the LPS-treated BV2 group. ^#^ denotes *p* < 0.05 compared with the corresponding GLS1 inhibitor group without α-KG, *n* = 5 per group

GLS1-mediated glutamine metabolism produces glutamate and subsequently α-ketoglutarate (α-KG). To determine whether α-KG is required for EV biogenesis and release, 1 mM of α-KG was added to LPS-stimulated BV2 cells with GLS1 inhibitors. Treatment with BPTES and CB839 significantly reduced GLS1 activity in the presence of 1 mM α-KG (Fig. 6a), confirming that they are indeed GLS1 inhibitors. In the presence of α-KG, BPTES and CB839 failed to decrease EV markers Alix and flotillin-2 in EV lysates (Fig. 6b-d). Consistent with the Western blots, NTA revealed that suppression of EV release by BPTES and CB839 was reversed by α-KG (Fig. 6e, f). These results suggest that α-KG is the critical downstream effector that is responsible for the GLS1-mediated EV release.

### GLS1 mediates EV release through sphingolipid metabolism

**Fig. 7:**
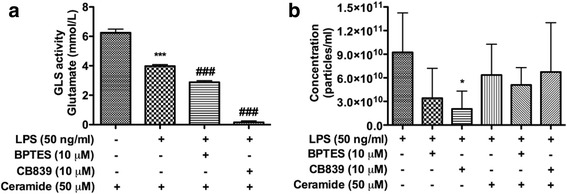
EV release in immune-activated microglia is associated with sphingolipid metabolism. BV2 cells were treated with 10 μM BPTES or 10 μM CB839 4 h prior to LPS treatment overnight. BV2 cell regular medium was changed to serum-free DMEM medium when GLS1 inhibitors were added. Two hours prior to LPS treatment, 50 or 100 μM ceramide was added to BV2 cells. **a** Protein lysates were collected from LPS-treated BV2 cells with or without GLS1 inhibitors or ceramide. GLS1 activities were determined by the enzyme activity assay. ANOVA and Bonferroni post-test; *** denotes *p* < 0.001 compared with that of the ceramide group. ^###^ denotes *p* < 0.001 compared with that of the LPS-treated group, *n* = 3 per group. **b** After 50-μM ceramide treatment, NTA was performed on the EVs isolated from the culture supernatants. Quantifications of the EV concentration were performed by NanoSight. ANOVA and Bonferroni post-test; * denotes *p* < 0.05 compared with that of the LPS-treated group, *n* = 5 per group

Our previous report has shown that the release of EV can be blocked by GW4869, a nSMase inhibitor [6, 20]. nSMase is known to catalyze the production of ceramide, which is an active component of EVs. Therefore, we hypothesized that GLS1 mediates EV release through sphingolipid metabolism. GLS1 activity remained suppressed by BPTES and CB839 in the presence of C_6_ ceramide (Fig. 7a), a cell-permeable short-chain ceramide that can be supplied to cell cultures as exogenous ceramides to increase cell ceramide levels [21]. However, NTA showed that BPTES- and CB839-mediated inhibition of EV release was blocked by C_6_ ceramide (Fig. 7b), indicating that the ceramide pathway of sphingolipid metabolism is involved in the GLS1-regulated release of EVs.

### Brain-specific GLS1 overexpression increases EV release in vivo

**Fig. 8:**
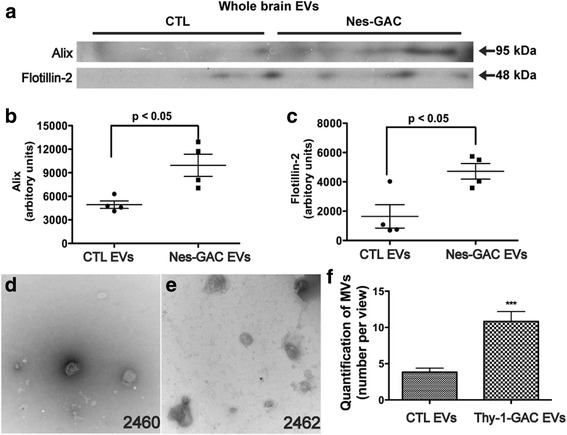
Brain-specific GLS1 overexpression increases EV release in vivo*.* **a** Wild-type and Nestin-GAC mice were sacrificed at 12–16 weeks old. The brains were removed, and for each brain, the right hemibrain was processed for EVs isolation and the left hemibrain was homogenized for brain lysates. Protein lysates were prepared from the EVs pellets in wild-type and Nestin-GAC hemibrains. The levels of EVs markers, tTG and flotillin-2, in the EVs lysates were analyzed by Western blot. EV protein loading was normalized with the weight of their corresponding hemibrains before the EV isolation. **b**, **c** Densitometric quantifications of the protein levels were presented as fold changes relative to that in wild-type EV lysate. Unpaired *t* test, *n* = 4 per genotype. Error bars are means ± S.E.M. **d**–**f** The brain tissues from adult Thy1-GAC mice were dissected, and EVs were isolated using the same techniques described above. EVs collected from WT (**d**) and Thy1-GAC (**e**) were evaluated via negative staining under TEM. Images shown were representative of each group. EV numbers per vision fields (*n* = 10) were quantified. CTL, control. Unpaired *t* test, *** denotes *p* < 0.001

To determine the link between the GLS1 and the secretion of EVs in vivo, we used a GLS1 transgenic mouse model that we recently generated [17]. The transgenic mice have GLS1 isoform GAC overexpressed in all cells of the neural lineage. Positive mice were termed Nestin-GAC transgenic mice. We successfully isolated EVs from the Nestin-GAC transgenic brain tissues following the protocol from a previous publication [22]. After normalized EVs to equal amount of initial brain tissues used for EV isolation, we found that Nestin-GAC mice had significantly higher levels of EV markers Alix and Flotillin-2 in the isolated EVs compared with those from control mice, suggesting that brain-specific overexpression of GLS increases EV release in vivo (Fig. 8a–c). To further characterize the EVs isolated from in vivo tissues, Thy1-GAC and wild-type (WT) were generated by crossing Thy1-Cre mice and CAG-loxp-GAC mice, and PCR reactions were performed to confirm the genotypes of the mice. EVs were isolated and purified from the hemibrains of both WT and Thy1-GAC mice and subjected to negative staining TEM analysis. The number of EVs was significantly higher compared with those in WT control (Fig. 8d–f). Together, these data indicate that the brain-specific GAC overexpression increases EV release in vivo.

## Discussion

Glutamine is the most abundant amino acid in the plasma, and its metabolic products provide energy and substrates for a variety of biosynthesis pathways. During viral infection and inflammation, glutamine metabolism is particularly important in that energy, biosynthesis, and antioxidative capacity are essential for a proper immune response. Our previous studies demonstrated a strong link between GLS and the neuropathogenesis of HIV-1 infection via the overproduction of neurotoxic levels of glutamate [11, 23–27]. Interestingly, GLS1 has been identified as an important metabolic factor controlling EV release from astrocytes in the presence of TNF-α [16]. However, it remains unclear how glutamine metabolism regulates EV biogenesis and release. The current studies present a major finding regarding EV release. Upregulation of GLS1 induces an increase in the release of EVs through α-KG and ceramide in HIV-1-infected macrophages and immune-activated microglia. The release of EVs is also observed to be increased in GAC-overexpressing transgenic mice. These new mechanistic regulations may help understand how glutamine metabolism shapes EV biogenesis and release during neuroinflammation.

Dysregulation of GLS1 has been reported in the pathogenesis of HIV-associated neurocognitive disorders and in cancer. In the CNS, GLS1 is a key enzyme in the glutamine metabolism, where glutamate, the main excitatory neurotransmitter, is produced to generate glutamate signaling and synaptic plasticity [28–32]. The dysregulation of GLS1 could potentially lead to the aberrant release of glutamate and compromise its neurotransmission. Indeed, abnormal glutamate neurotransmission is strongly associated with the memory loss and learning deficits due to the disrupted functioning of NMDA receptors [31–33]. To model upregulation of GLS1, we first constructed new adenoviruses overexpressing GLS1 isoforms. KGA- and GAC-overexpressing HeLa cells release a higher number of EVs into the supernatants. KGA- and GAC-mediated EV release can be blocked by GLS1 inhibitors BPTES and CB839, suggesting GLS1 is a critical factor regulating EV biogenesis and release. Similarly, we also used HIV-1-infected macrophages and LPS-treated microglia to model GLS1 upregulation. HIV-1-infected macrophages and LPS-treated microglia have higher GLS1 levels compared to those of controls, which are associated with higher levels of EVs. Our third approach to model GLS1 upregulation is to use newly generated GAC transgenic mice. More EVs can be collected from GAC transgenic mice compared with those from negative littermates. However, it remains to be confirmed whether GLS1 inhibitors can block the EV release in vivo and whether the blocking of EV release could have protective effects on the GAC transgenic mice.

Our investigations reveal potential mechanisms of EV release in the context of HIV-1 infection and neuroinflammation. First, the release of EVs is dependent on the presence of glutamine. Second, α-KG, a downstream product of glutamate, can rescue the inhibition of EV release by GLS1 inhibitors. Third, C_6_ ceramide, a cell-permeable analog of ceramide, can rescue the inhibition of EV release by GLS1 inhibitors. In glutamine repletion experiments, changes in EV release were observed at as low as 1 mM of glutamine, the level of which is close to plasma concentration of glutamine [34]. However, the increase of glutamine concentration from 1 to 5 mM did not show a dose-dependent increase of EVs. Further testing of lower concentrations of glutamine will help to establish a dose-dependent effect of glutamine. Another limitation of the current studies involves the experiments on α-KG and ceramide. It is unknown whether the effect of α-KG and C_6_ ceramide on EV release was direct or through other downstream metabolic intermediates. Given the variety of metabolic intermediates downstream of α-KG, it is likely that other contributing factors in this pathway are required for EV biogenesis and release. Indeed, recent data suggest specific mechanisms controlled by glutaminolysis to fine-tune macrophage activities during both M2 and LPS activation involve a-KG and succinate [35, 36]. EV biogenesis and release from macrophages may aid in the macrophage-mediated immune responses to infection. Therefore, harnessing EVs through glutamine/a-KG pathway would be an attractive strategy to regulate macrophage phenotypes in infection and inflammation.

Our characterizations of EVs involve the determination of EV markers in Western blots, EV size/concentrations in well-established NanoSight, and the morphological examination through negative staining TEM. Notably, the size of EVs remains the same throughout all the studies as determined by NanoSight analysis, indicating that the regulation of the EVs during HIV-1 infection and immune activation of macrophages/microglia are more specifically on the number and contents. EVs are known to include exosomes, microvesicles, and apoptotic bodies according to their cellular origin. In our studies, there is little evidence of apoptotic bodies in the EVs since the size of EVs was overwhelmingly smaller than 300 nm, whereas typical apoptotic bodies are more than 500 nm. Furthermore, it is known that EV isolation contains virions, and HIV-1 virions are around 145 nm [37], which may be mistaken as EVs during data interpretation. However, based on the data from NanoSight, EV quantities (10^8^ ~ 10^10^/ml) greatly exceed the quantity of viral particles (~ 10^6^/ml) in the supernatant. Therefore, we conclude that the particles detected by the NanoSight are predominantly EVs, including exosomes and microvesicles.

Previously, we reported that using GW4869 could effectively inhibit the release of EVs in HIV-1-infected macrophages and LPS-treated microglia [6]. Studies have also shown that GW4869 could inhibit EV release in vitro and in vivo in AD models and prion diseases [3, 7, 38–42]. It has also been reported that sphingolipid metabolism could be involved in EV release in microglia and neurological diseases [5, 43]. Our data demonstrate a causal role of ceramide in mediating GLS1-induced EV release. However, the exact role of sphingolipid metabolism in GLS1-associated EV release remains to be fully elucidated. It is unclear whether other lipid pathways also involved in GLS-mediated EV regulation [44, 45]. Whether sphingolipid metabolism merely provides building blocks for EVs or more refined mechanisms are involved remains to be determined.

## Conclusions

In summary, our studies suggest that GLS1-mediated glutamine metabolism is essential in regulating EV release during HIV-1 infection and immune activation. GLS1 regulates EV release through its key downstream products α-ketoglutarate and ceramide. GLS1 overexpression in the brain leads to increased levels of EVs in vivo. These new mechanistic regulations may help understand how glutamine metabolism shapes EV release during infection and inflammation. Targeting glutamine/a-KG pathway would be an attractive strategy to regulate EV release.

## Methods

### Ethics statement

MDM were used in full compliance with the University of Nebraska Medical Center and National Institutes of Health ethical guidelines, with the Institutional Review Board (IRB) #: 162-93-FB. We have the informed written consent from all participants involved in this study. All mice were housed and bred in the Comparative Medicine Animal Facilities at the University of Nebraska Medical Center. All procedures were conducted in accordance with the protocol (11-018-04) approved by the Institutional Animal Care and Use Committee at the University of Nebraska Medical Center.

### Culture, HIV-1 infection, and LPS activation of macrophages and microglia

Human peripheral blood-derived mononuclear cells were isolated through leukopheresis from healthy donors. Human macrophages were differentiated in Dulbecco’s modified Eagle’s media (DMEM) (Sigma-Aldrich, St. Louis, MO) with 10% human serum, 50 μg/ml gentamycin, 10 μg/ml ciprofloxacin (Sigma), and 1000 U/ml recombinant human macrophage colony-stimulating factor (MCSF) for 7 days. Human fetal microglial cells were obtained from fetal brain tissue-derived microglia-astrocytes mixed cultures as previously described [46]. The HIV-1_ADA_ strain was used to infect the macrophages and microglia at a multiplicity of infection (MOI) of 0.1 and 0.5, respectively. After 24 h, the culture medium was changed to remove any remnant virus. Seven days after HIV-1-infection, the culture medium was changed to glutamine-free neurobasal medium for 24 h, and supernatants were collected for subsequent HPLC or Western blot analysis. HeLa and BV_2_ cell lines were obtained from ATCC, and both cell lines were grown in DMEM with 10% fetal bovine serum and antibiotics. LPS was used to immune activate BV_2_ cells for 24 h, and supernatants were collected for HPLC and Western blot analysis. Bis-2-(5-phenylacetamido-1,2,4-thiadiazol-2-yl)ethyl sulfide (BPTES) and CB839 (generous gifts provided by Dr. Takashi Tsukamoto from John Hopkins University and later ordered from Millipore with catalog numbers 530030 and 533717, respectively) were used in HIV-1-infected macrophages or LPS-treated microglia prior to EV isolation. α-Ketoglutarate (Sigma; 349631) and C_6_ ceramide (Sigma; H6524) were also used to manipulate the metabolic intermediates in HIV-1-infected macrophages or LPS-treated microglia. All experiments involving human cell samples are approved by the Institutional Review Board at the University of Nebraska Medical Center.

### Adenoviral constructs

Replication-defective adenovirus vectors expressing human KGA and GAC were generated using RAPAd® CMV Adenoviral Expression System (Cell Biolabs, Inc., San Diego, CA). Generation of the full-length human KGA and GAC constructs was described in our prior publication [12]. Adenoviral constructs were amplified in a 293 AD cell line (Cell Biolabs) and purified by ultracentrifugation through a CsCl gradient. Viral titer was determined by the Adeno-X™ Rapid Titer Kit (Clontech Laboratories, Inc., Mountain View, CA).

### Isolation of EVs from cells

EVs were isolated from the supernatants of GLS1-overexpressing cells, HIV-1-infected macrophages, and LPS-activated microglia through differential centrifugations. Briefly, the supernatants were first centrifuged at 300×*g* for 10 min to remove free cells, at 3000×*g* for 20 min to remove cellular debris, and then 10,000×*g* for 30 min to remove free organelles. Lastly, EVs were collected by ultracentrifugation at 100,000×*g* for 2 h at 4 °C. To prepare EVs for Western blotting, the EV pellets were lysed in M-PER mammalian protein extraction reagent (Thermo Scientific, Pittsburgh, PA). For negative staining, EVs were fixed in 2% glutaraldehyde and 2% paraformaldehyde. For glutaminase activity assay and neurotoxicity, the EVs were resuspended in 1 ml of glutamine-free neurobasal medium.

### Isolation of EVs from mice brain

EV isolations from the brains were carried out as described previously with modifications according to the protocol [22]. The fresh and previously frozen mice hemibrains were harvested and dissected finely. The brain samples were then treated with 20 units/ml papain (Worthington) in Hibernate E solution (BrainBits, Springfield, IL) for 15 min at 37 °C. The same volume of cold Hibernate E solution was added to the brain samples to stop the reaction of papain. The brain tissue was then gently homogenized and filtered through a 40-μm mesh filter (BD Biosciences), followed by a centrifugation at 300×*g* for 10 min and 3000×*g* for 20 min at 4 °C to get rid of cells, membranes, and debris. After the supernatants were filtered through 0.45-μm filter (Thermo Scientific), they were subjected to 10, 000×*g* for 30 min at 4 °C to eliminate organelle contaminations. The supernatants were further centrifuged at 100,000×*g* for 70 min at 4 °C to pellet EVs. The pellets were then resuspended in filtered PBS, or MPER lysate solution for NanoSight or Western blot. All the samples were ultracentrifuged in ultraclear polycarbonate tubes (Beckman Coulter) that have a volume of 13.2 ml. A Beckman Coulter ultracentrifuge (Beckman Coulter OptimaL-90K ultracentrifuge; Beckman Coulter, Fullerton, CA, USA) was used with a rotor type SW 41 Ti.

### Negative staining and electron microscopy

EVs were fixed and then spread on the silicon monoxide and nitro-cellular film-coated copper grid. The droplets were removed with filter paper, air-dried at room temperature, and then subjected to transmission electron microscopy (TEM).

### Nano-particle tracking analysis

A NanoSight NS 300 (Malvern) equipped with an sCMOS camera was utilized to analyze the size distribution and concentration of EVs. NanoSight utilizes NTA, which is a combination of light scattering and Brownian motion technology to measure the concentration and size and distribution of particles in the EV supernatants. After the whole process of EV isolation, the pellets were first resuspended in 100 μl of filtered PBS and then diluted 100 times. The conditions of the measurements include temperature of 25 °C; viscosity of 1 cP, 25 s per capture frame; and a measurement time of 60 s. All the conditions were kept the same among all the samples. The results indicate the mean sizes and concentration of at least three individual measurements.

### Western blot

Protein concentrations were determined by Bradford protein assay. SDS PAGE separated proteins from the whole cell and EV lysates. Afterward, they were electrophoretically transferred to polyvinyldifluoridene membranes (Millipore, Billerica, MA and Bio-Rad, Hercules, CA). The membranes were incubated overnight at 4 °C with polyclonal antibodies for KGA and GAC (Dr. N. Curthoys, Colorado State University, Fort Collins, CO), tissue transglutaminase (tTG) (Lab Vision/Thermo, Fremont, CA), flotillin-2 (Cell Signaling Technology, Danvers, MA), and β-actin (Sigma), followed by horseradish peroxidase-linked secondary anti-rabbit or anti-mouse secondary antibodies (Cell Signaling Technology). Antigen-antibody complexes were visualized by Pierce ECL Western Blotting Substrate. For quantification of the data, films were scanned with a CanonScan 9950F scanner, and images were analyzed using the public domain NIH Image program (developed at the US National Institutes of Health).

### Glutaminase activity assay

Highly concentrated whole cell lysates were collected from flasks and subjected to GLS activity assay using a two-step assay [27, 47]. Briefly, protein concentrations in the lysates were tested by using BCA Protein Assay Kit (Pierce). All samples were normalized to same concentration. In the first step, 50 mg of protein were added to 100 μl of initial assay mix. The mix contains 50 mM glutamine, 0.15 M phosphate, 0.2 mM EDTA, and 50 mM Tris-acetate. The PH value of the mix was adjusted to 8.6 and incubated at 37 °C for 30 min. Ten microliters of 3 N hydrochloric acid was added to inactivate the glutaminase activity and stop the reaction. In the second step, 1 ml of the second reaction mix was added, which contained 0.4 mg of purified bovine liver glutamate dehydrogenase (Sigma-Aldrich, St. Louis, MO, USA), 0.08 M Tris-acetate at pH 9.4, 0.2 M hydrazine, 0.25 mM adenosine 5′-diphosphate sodium salt, and 2 mM β-nicotinamide adenine dinucleotide hydrate. The samples were mixed and incubated for 30 min at room temperature. One hundred microliters of the reaction was used for measurement, absorbance was determined at a wavelength of 340 nm, and glutamate concentration was determined using a standard curve of 10, 5, 2.5, 1.25, 0.625, and 0.0 mM glutamate, along with negative controls.

### Analysis of glutamate concentrations

Glutamate levels were analyzed by RP-HPLC using an Agilent 1200 liquid chromatograph and fluorescence detector as previously described [14] with a few modifications. The experiments utilized 4.6 × 75 mm, 3.5 μm ZORBAX Eclipse AAA analytical columns (Agilent). A gradient elution program was optimized for glutamate measurement with a flow rate 0.75 ml/min. The intracellular glutamate levels in the whole brain lysates of mice and whole cell lysates were determined by Amplex Red Glutamic Acid/Glutamate Oxidase Assay Kit (Invitrogen) based on the manufacturer’s instruction. The brain tissue lysates and whole cell lysates were diluted to the same protein concentration before the assay.

### Statistical analysis

Data are expressed as means ± SD unless otherwise specified. Statistical analysis was performed using one-way analysis of variance (ANOVA), followed by the Bonferroni post-test for all paired observations unless otherwise specified. Significance was determined by a *p* value < 0.05. All experiments were performed with cells from at least three donors to account for any donor-specific differences. Assays were performed at least three times in triplicate or quadruplicate within each assay.

## Additional file

Additional file 1: **Figures S1.** Both KGA and GAC are successfully overexpressed by adenovirus in vitro. S2: EV release in HIV-1-infected macrophages is dependent on glutamine. S3: LPS, BPTES, and CB839 do not affect BV2 cell viability. (DOCX 6796 kb)

## Abbreviations

Alix: ALG-2 interacting protein
ANOVA: Analysis of variance
BPTES: Bis-2-(5-phenylacetamido-1,3,4-thiadiazol-2-yl)ethyl sulfide
CNS: Central nervous system
EV: Extracellular vesicle
GAC: Glutaminase C
GLS1: Glutaminase 1
KGA: Kidney-type glutaminase
LPS: Lipopolysaccharide
MOI: Multiplicities of infection
nSMase: Neutral sphingomyelinase
NTA: Nanoparticle tracking analysis
RP-HPLC: Reversed-phase high-performance liquid chromatography
TEM: Transmission electron microscopy
tTG: Tissue transglutaminase
α-KG: α-Ketoglutarate
