# Supplementary material for: Glutaminase 1 regulates the release of extracellular vesicles during neuroinflammation through key metabolic intermediate alpha-ketoglutarate
Source: J Neuroinflammation. 2018 Mar 14;15:79. doi: 10.1186/s12974-018-1120-x (PMC5853116; doi:10.1186/s12974-018-1120-x)
Supplement: Supplementary file 1 — Figures S1. Both KGA and GAC are successfully overexpressed by adenovirus in vitro. S2: EV release in HIV-1-infected macrophages is dependent on glutamine. S3: LPS, BPTES, and CB839 do not affect BV2 cell viability. (DOCX 6796 kb) [file 12974_2018_1120_MOESM1_ESM.docx]

**Supplementary Material**

**Fig. S1. Both KGA and GAC are successfully overexpressed by adenovirus in vitro.**


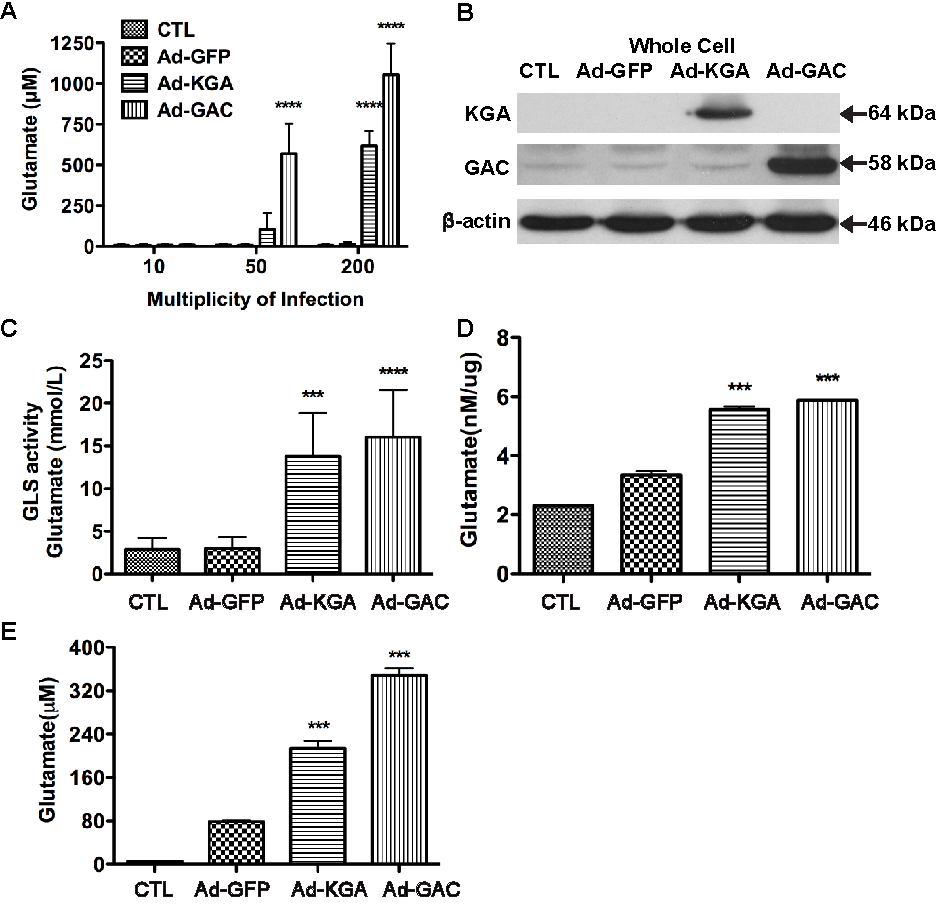


**(A)** Cell-free supernatants were collected from adenovirus-infected HeLa cell cultures at the MOI of 10, 50 and 200. RP-HPLC were used to determine the extracellular glutamate level. **(B)** GLS1 was overexpressed through adenovirus vectors that express KGA and GAC isoforms in HeLa cells at a multiplicity of infection (MOI) of 200. Two days after adenovirus infection, proteins lysates were collected from whole cells and the levels of KGA, GAC, tTG and flotillin-2 were determined by Western blot. Actin was used as loading control. **(C)** Protein lysates were collected from KGA- and GAC-overexpressing HeLa cells at the MOI 200. GLS1 activities were determined by the enzyme activity assay. GFP adenovirus Ad-GFP was used as vector control. **(D, E)** Extracellular and intracellular glutamate levels from adenovirus-KGA and -GAC infected HeLa cells with MOI 200 were detected by Amplex Red Glutamic acid/Glutamate oxidase Assay Kit and RT-HPLC, respectively. Quantification results shown are means ± SD of experiments performed in triplicate (n = 3 donors). CTL, control. *** and **** denotes p < 0.001and 0.0001, compared with the Ad-GFP group.

**Fig. S2. EV release in HIV-1-infected macrophages is dependent on glutamine.**


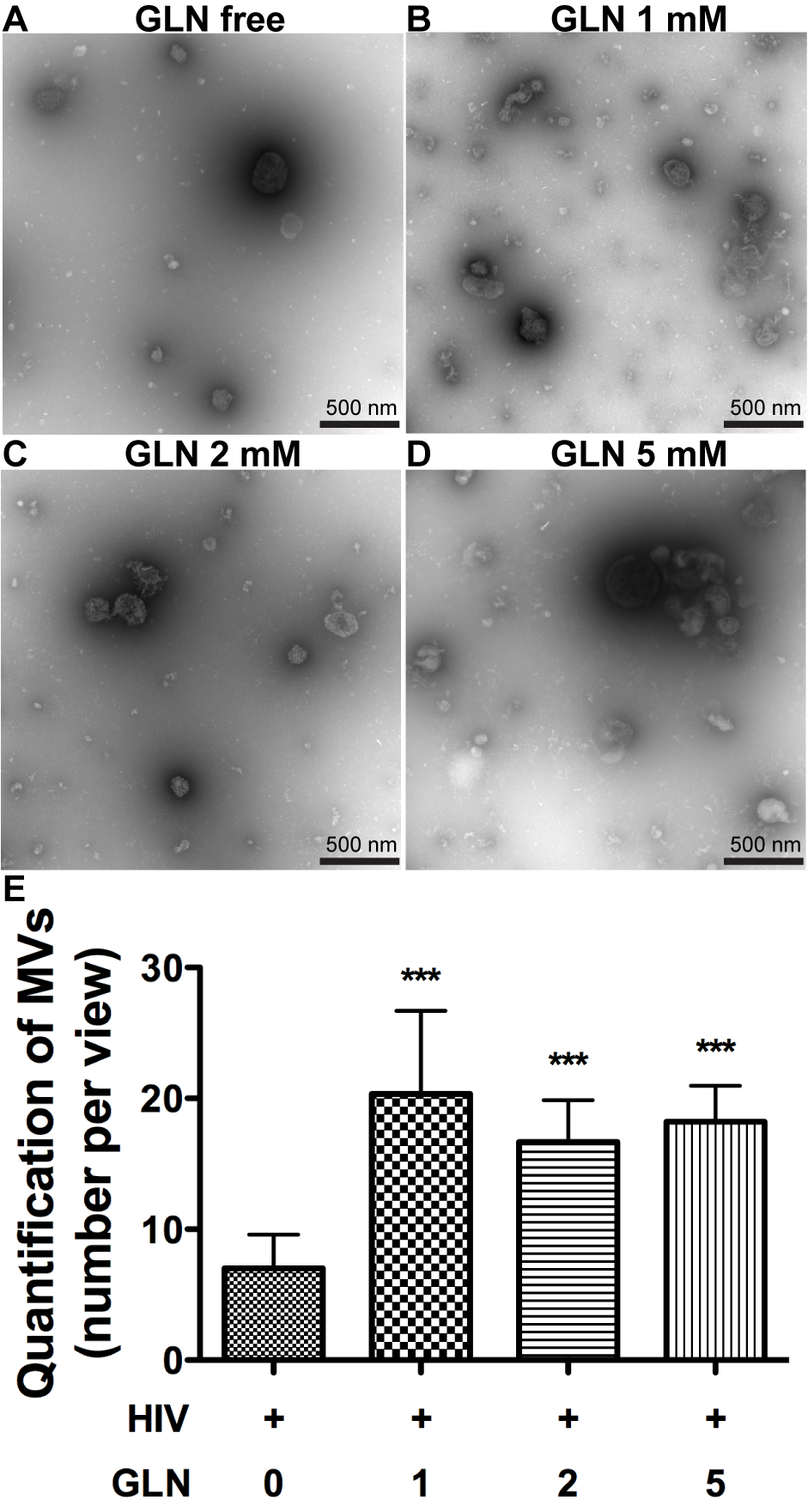


**(A-D)** MDM was infected by HIV-1 virus for 6 days, and the medium was changed into serum free glutamine-free DMEM. Additional glutamine was added to the medium at the concentrations of 1 mM, 2 mM and 5 mM. EVs were isolated through differential centrifugation and resuspended upon the concentrations of whole cell lysates. EVs were fixed and subjected to negative staining using TEM under a magnification of 42,000 X (E-H). **(E)** EVs numbers in E to H were quantified by manually counting from a total of 7 random vision fields. Results are representative of TEM images and quantification results are means ± SD of EV numbers from 7 fields of TEM images. *** denotes p < 0.001 in comparison to controls.

**Fig. S3. LPS, BPTES, and CB839 do not affect BV2 cell viability.**

**
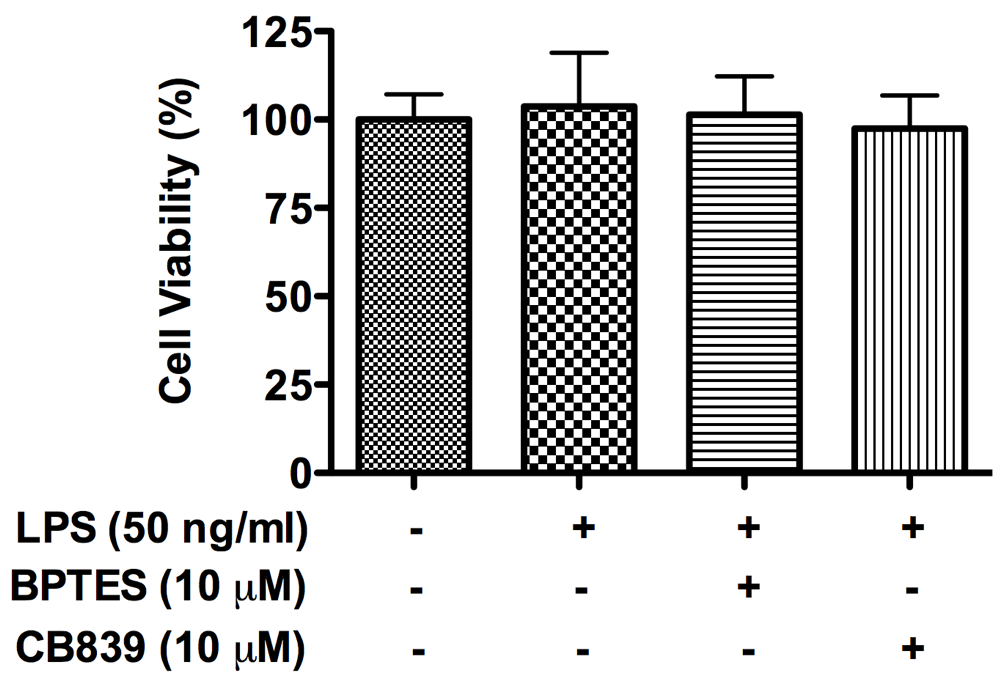
**

BV2 cells were treated with 10 µM BPTES or 10 µM CB839 4 hours prior to LPS treatment overnight as outlined in Figure 5A. At the experimental end point, cell viability was determined by a colorimetric MTS assay CellTiter 96^®^ AQueous One Solution Assay (Promega, Madison, WI) based on the manufacture’s instruction. Results were normalized as percentage of cell viability in control BV2 cells.
